# Long-Term Compressive Behavior Evaluation for SSC and Prediction Using Machine Learning Models with SHAP Interpretation

**DOI:** 10.3390/ma19204423

**Published:** 2026-10-21

**Authors:** Qinghai Xie, Jiale He, Qucheng Xu, Zheming Wen, Jie Zeng, Zhongling Zong

**Affiliations:** 1 School of Civil and Ocean Engineering, Jiangsu Ocean University, Lianyungang 222005, China; 2024220405@jou.edu.cn (J.H.); 2024220427@jou.edu.cn (Q.X.); 2023220428@jou.edu.cn (Z.W.); 2023220401@jou.edu.cn (J.Z.); jouzongzhl@jou.edu.cn (Z.Z.); 2 Department of Structural Engineering, Tongji University, Shanghai 200092, China

**Keywords:** seawater sea-sand concrete, compressive behavior, machine learning, BP neural network, predictive modeling

## Abstract

This study investigates the time-dependent compressive behavior of seawater sea-sand concrete (SSC) and establishes intelligent prediction models for its evaluation. SSC samples with three strength grades (C30, C40, and C60) were fabricated and tested at curing ages of up to 720 d, accompanied by microstructural characterization. A database containing 344 compressive-strength records was compiled from the present experiments and published literature. Four models were developed and compared, including a back-propagation (BP) neural network, a genetic algorithm-optimized BP (GA-BP) network, a Bagging-BP ensemble model, and a gradient boosting regression tree (GBRT) model. Model interpretability was further examined using SHapley Additive exPlanations (SHAP). The results indicate that SSC exhibits rapid early-age strength gain, while moderate fluctuations may occur during middle and later curing periods. Strength–elastic modulus relationships were established for SSC and showed satisfactory fitting accuracy, with R^2^ values exceeding 0.80. Among the investigated models, GA-BP demonstrated favorable predictive performance under the adopted data split, achieving the lowest root mean square error with R = 0.9413. SHAP interpretation highlighted curing duration and water-to-cement ratio as the dominant factors governing compressive strength. Overall, the proposed GA-BP model can serve as a useful reference for SSC strength prediction and mix-design optimization within the current dataset.

## 1. Introduction

In recent years, the development of concrete materials for marine infrastructure has received increasing research interest. Seawater sea-sand concrete (SSC) is produced by incorporating seawater and sea sand directly into concrete mixtures, thereby reducing the dependence on freshwater and river sand in coastal and island regions [1]. Previous studies have reported that the presence of marine raw materials can promote early-age strength development in SSC [2]. However, its long-term behavior has not yet been fully clarified, especially with respect to hydration-product evolution, pore-structure variation, and mechanical-property development. These uncertainties may limit the reliable use of SSC in practical engineering applications [2,3,4].

Previous studies have reported that the ions and fine particles introduced by seawater and sea sand may interact with the cementitious matrix during hydration, leading to changes in hydration reactions and pore-structure development. As a result, the strength evolution of SSC is closely related to curing age [5,6]. At early ages, accelerated hydration and pore refinement can promote matrix densification and rapid strength gain. However, at later ages, strength development may exhibit instability due to chloride binding, ettringite formation, and changes in the morphology of C–S–H [7]. These age-related microstructural processes contribute to the nonlinear development of the mechanical performance of SSC, thereby limiting the accuracy of conventional empirical equations in describing its long-term strength behavior.

In materials science, data-driven modeling has become an effective tool for analyzing the mechanical behavior of concrete, particularly when multiple mixture parameters interact in a nonlinear manner. Compared with conventional empirical formulas, machine learning-based approaches can extract hidden relationships from experimental datasets and improve the reliability of strength estimation [8]. For example, Javid et al. [9] compared several learning algorithms, including tree-based methods, to establish a framework for concrete compressive-strength prediction. Zhang et al. [10] demonstrated that random forest regression can provide reliable estimates of the uniaxial compressive strength of lightweight self-compacting concrete. For self-compacting concrete containing recycled coarse aggregate. Jagadesh et al. [11] investigated self-compacting concrete mixtures containing recycled coarse aggregates and developed ANN-based computational models to estimate their compressive strength. In addition, Hamed et al. [12] further improved prediction accuracy by developing a stacked model that integrated ANN, RF, and XGBoost. Recent studies have also extended machine-learning approaches to concrete mixed with seawater or sea-sand. Yu et al. [13] developed an ANN model for predicting the 28 d compressive strength of SSC based on the water-to-cement ratio, cement content, and sand ratio. De Alwis et al. [14] applied ANN-based prediction to self-compacting concrete mixed and cured with seawater, while Zhu et al. [15] combined multiple machine-learning models with SHAP analysis to predict and interpret the compressive strength of sea-sand concrete.

Against this background, establishing a reliable data-driven approach that links SSC strength performance with mixture parameters is essential for engineering design and structural safety evaluation. Most conventional empirical equations were established on the basis of ordinary concrete theory and therefore are inadequate for accurately describing the nonlinear mechanical behavior of SSC. Consequently, data-driven modeling has gradually become a major research focus. In particular, machine-learning-based prediction methods have become an important tool for concrete performance analysis, as they are capable of describing nonlinear relationships among various influencing parameters [16]. Among various methods, back-propagation (BP) neural networks have been frequently adopted for their effectiveness in modeling complex material relationships [8,17]. To mitigate the tendency of BP networks to converge to local optima and their sensitivity to initial parameters, hybridization strategies such as genetic algorithm (GA) optimization have been shown to enhance global search capability and prediction stability [18]. Meanwhile, ensemble methods such as Bagging can reduce the sensitivity of a single model to noise and improve generalization performance [19]. In addition, gradient boosting regression trees (GBRT), a rapidly developing class of Boosting-based methods in recent years, have demonstrated substantial potential in material-property prediction because of their strong ability to model feature interactions and their high predictive accuracy [8].

This study focuses on the long-term development of mechanical properties of SSC, investigating its mechanical performance and associated microstructural evolution. Following our previous study [20] which examined the SSC up to 150 days, this study further investigates its performance up to 720 days. A predictive framework for compressive strength is established by incorporating key mixture proportions and curing age into four models: BP, GA-BP, Bagging-BP, and GBRT. Furthermore, the SHAP approach is adopted to reveal the prediction mechanism of the models and to assess the relative influence of individual input variables. By comprehensively evaluating fitting accuracy, generalization capability, and feature importance, this work aims to provide an efficient method for assessing SSC performance and a reliable basis for strength prediction, thereby supporting its wider application in marine engineering.

## 2. Materials and Methods

### 2.1. Raw Materials

**Figure 1:**
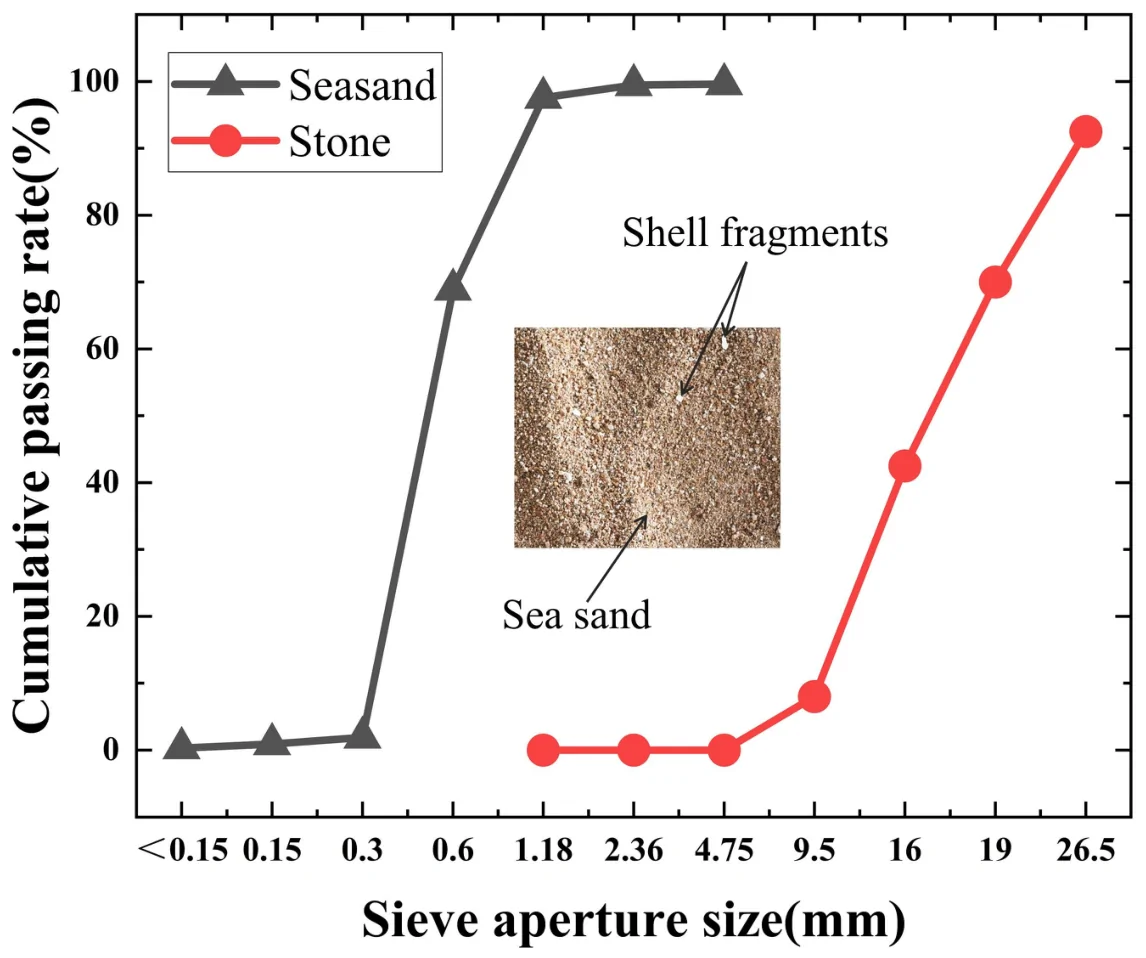
Cumulative passing percentage.

Natural seawater and sea sand collected from the coastal area of Haizhou Bay in the Yellow Sea, China, were used in this study. The sulfate ion and chloride ion concentrations in the seawater were 1.7 g/L and 14.0 g/L, respectively. Before mixing, visible shell fragments and other foreign substances in the sea sand were removed through a 5 mm sieve, and its fineness modulus was determined to be 2.31. The physical characteristics of the sea sand are listed in Figure 1 and Table 1 [21].

Crushed stone with particle sizes ranging from 5 to 20 mm, sourced from the Haizhou Sand and Gravel Market (Lianyungang, China), was adopted as the coarse aggregate. Its cumulative passing percentage is shown in Figure 1, and its crushing index was 16%. Ordinary Portland cement with strength grades of P.O. 42.5 and P.O. 52.5 (Zhucheng Yangchun Cement Co., Ltd., Zhucheng, China) was used for different strength-grade SSC mixtures, in which P.O. 42.5 was used for C30 and C40, while P.O. 52.5 was used for C60. A solid powdered polycarboxylate high-range water-reducing admixture was used (Chenqi Chemical Technology Co., Ltd., Shanghai, China).

### 2.2. Mix Proportions and Experimental Methods

The corresponding SSC mixtures were designed at three target strength grades, namely C30, C40, and C60. The detailed mixture parameters are listed in Table 2. All SSC specimens in this study were prepared using the same mix proportions as reported in our previous work [20], where compressive strength data were only available at 3, 7, 14, 21, 28, 60, and 150 curing days. In the present study, the same batches of specimens were continuously cured and tested at extended ages. Specifically, new compressive strength measurements were obtained for C30 at 240, 360, and 420 days; for C40 at 240, 360, 420, 600, and 720 days; and for C60 at 240 and 360 days. All data beyond 150 days presented herein are original and have not been previously published.

It should be noted that no reference concrete prepared with freshwater and river sand was included in the present experimental program. Therefore, the present experiments characterize the behavior of the investigated SSC mixtures rather than isolate the individual effects of seawater, sea sand, or any single mixture parameter.

SSC mixtures were produced in a compulsory horizontal-shaft mixer (Xianxian Hongwu Building Materials Processing & Sales Center, Hebei, China). The freshly mixed concrete was placed into cube molds with dimensions of 100 mm × 100 mm × 100 mm and prism molds with dimensions of 100 mm × 100 mm × 300 mm, followed by vibration to ensure adequate compaction. All SSC specimens were cured and tested following the same procedures as reported in our earlier study [20]. After demolding at 24 h, specimens were placed in a standard curing room and subjected to a short-term seawater spraying treatment for the first 7 days. During this period, natural seawater was sprayed onto the specimen surfaces twice daily, and the specimen surfaces were maintained in a wet condition between spraying cycles in the standard curing room (20 ± 2 °C, RH ≥ 95%). Afterwards, specimens continued to be stored in the standard curing room without further seawater treatment until the age of 28 days. All specimens were then transferred to the indoor laboratory environment and kept uncovered until the designated testing ages. The indoor temperature and relative humidity during this period were not systematically recorded.

**Figure 2:**
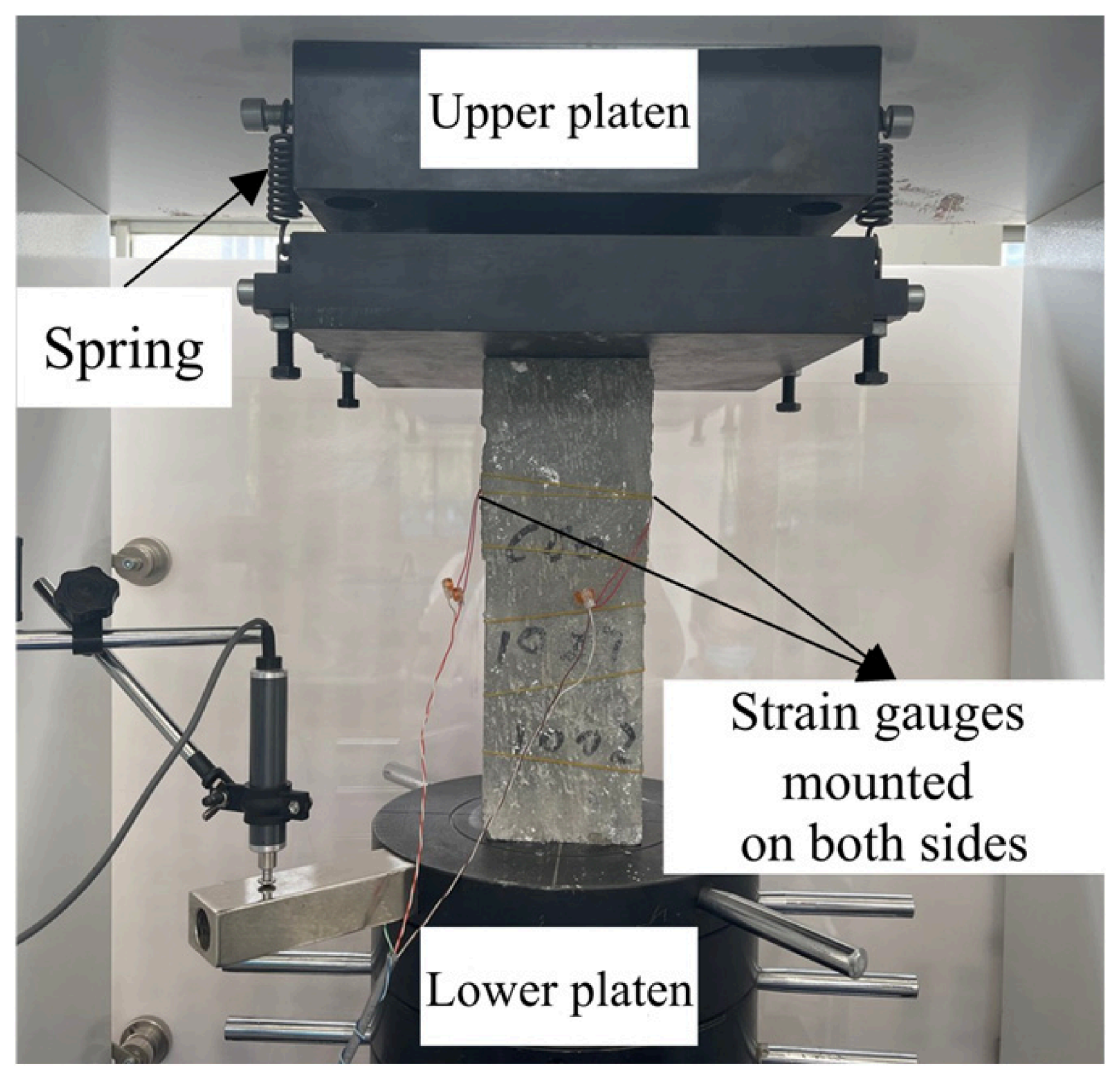
Concrete axial compression test.

For each strength-grade–age condition, three cube specimens were tested, and the reported compressive strength was calculated as the mean of the three measurements, with the standard deviation used to characterize the dispersion of the results. Accordingly, 30, 36, and 27 cube specimens were tested for the C30, C40, and C60 mixtures, respectively, giving a total of 93 cube specimens. In addition, a total of 18 prism specimens were tested at 360 d, with six specimens for each strength grade. For each grade, three specimens were used to determine the axial compressive strength *f*_c_, while the remaining three were used for the elastic-modulus test. The tests were conducted in accordance with GB/T 50081-2019 [22], with loading rates of 0.5, 0.8, and 1.0 MPa/s for C30, C40, and C60, respectively. The specimens were preloaded to 0.5 MPa for 60 s, then loaded to *f*_c_/3 and held for 60 s, and finally unloaded back to 0.5 MPa. This loading–unloading cycle was repeated twice. Subsequently, the specimens were loaded to *f*_c_/3 for 60 s, after which the load was increased monotonically until failure for deformation measurement. The test arrangement is shown in Figure 2.

### 2.3. Microstructural Tests

**Figure 3:**
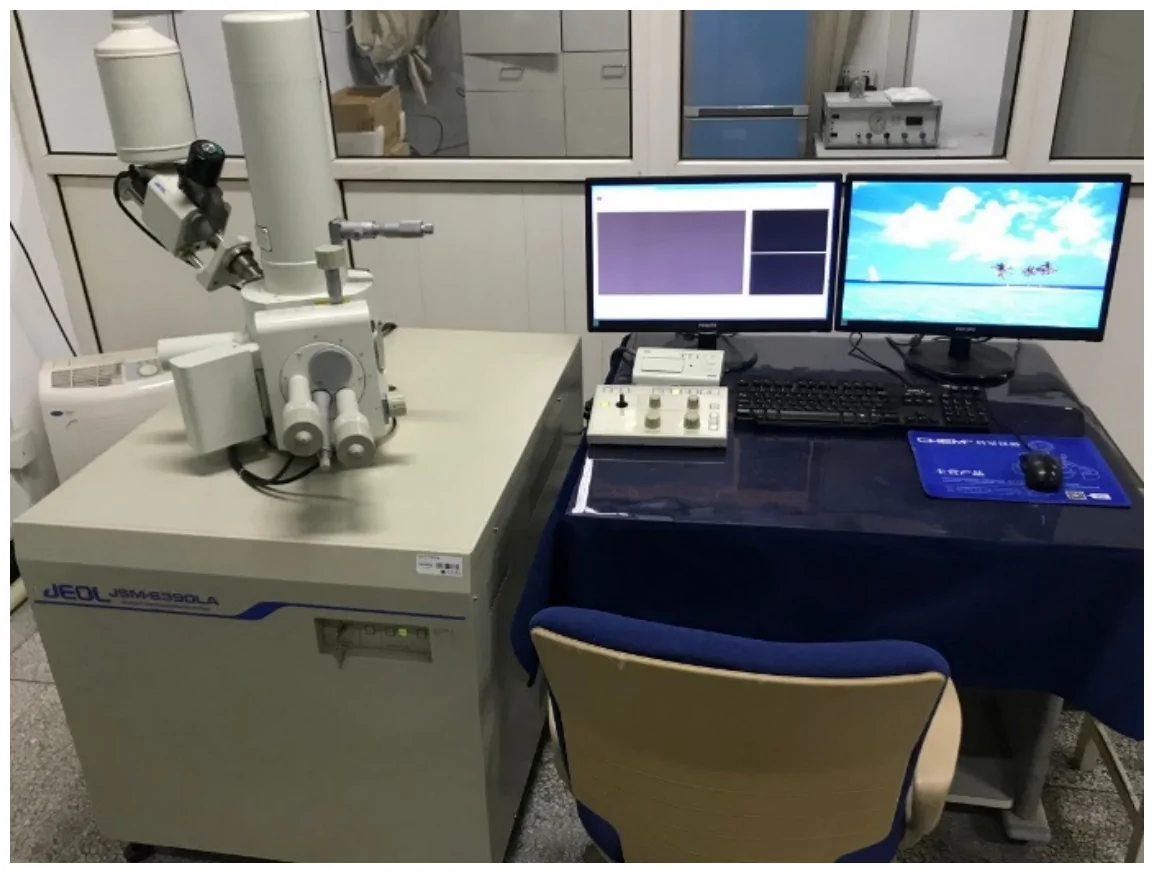
Scanning electron microscope.

Fragments of cement paste with dimensions of approximately 5 mm × 5 mm and relatively flat surfaces were selected from the broken specimens as samples for microstructural observation. The SEM specimens were collected at 28 d. Observations were then carried out using a JSM-6390LA scanning electron microscope (SEM; JEOL Ltd., Tokyo, Japan) (Figure 3). During image acquisition, an accelerating voltage of 20 kV was applied, and representative microstructural morphologies were obtained at different magnifications to reveal the evolution of the hydration structure of the material under the combined action of seawater and sea sand.

## 3. Machine Learning Model Development

### 3.1. BP Neural Network

The back-propagation (BP) neural network is a type of feedforward artificial neural network [23]. During model training, input data are propagated through the network layers to generate the predicted output. The prediction error is then used in the backward calculation process to update the connection weights and bias terms. This learning procedure is repeated until satisfactory convergence is achieved. BP neural networks have been widely used for estimating the mechanical properties of concrete made with construction and demolition waste [24]. However, such prediction models do not explicitly consider the failure mechanism of concrete [25].

In this study, the BP neural network was configured with one hidden layer containing 10 neurons and trained using the Levenberg–Marquardt algorithm (trainlm) for a maximum of 1000 epochs. The hidden and output layers employed tansig and purelin transfer functions, respectively. A 70:15:15 train/validation/test split and a fixed random seed of 2025 were adopted.

### 3.2. GA-BP Neural Network

The genetic algorithm (GA) is a population-based global optimization algorithm inspired by the principles of natural selection and biological evolution. Through iterative optimization, it can continuously improve the computational results and drive them closer to the global optimum [26]. Researchers used GA-based models to estimate the compressive behavior of thermal insulation concrete incorporating recycled aggregates [27] and ultra-high-performance concrete [28]. In the present study, the initial parameters of the BP neural network, including connection weights and bias terms, were optimized using GA to improve global searching efficiency and reduce the risk of premature convergence.

For GA-BP, an ensemble of 13 sub-models with hidden-layer sizes selected from 10, 12, and 14 neurons was constructed. The genetic algorithm used a population size of 90, a maximum of 130 generations, a crossover fraction of 0.80, and a weight-and-bias search range of [−3, 3], followed by BP refinement using trainbr for up to 1000 epochs. A 70:15:15 train/validation/test split and a fixed random seed of 2025 were adopted, and the final predictions were combined using non-negative least-squares weighting. The test set was kept independent of model optimization and ensemble weighting and was used only for final performance evaluation.

### 3.3. Bagging-BP Neural Network

The Bagging-BP neural network combines the bootstrap aggregating strategy with the BP neural network. In the Bagging-BP model, several BP neural networks are independently trained using different bootstrap samples, and their prediction results are averaged to generate the final output. This strategy reduces the instability of a single BP model and improves the generalization performance of the ensemble [29]. This method can effectively reduce model variance and mitigate the sensitivity of a single network to sample variation and initial weights, thereby improving prediction stability and generalization ability in material-property prediction [30].

The Bagging-BP framework consisted of 15 BP base learners trained on bootstrap samples generated with replacement from the training set. Each learner contained one hidden layer with 4, 6, 8, or 10 neurons and was trained using Bayesian regularization (trainbr) for up to 1200 epochs. A 70:15:15 train/validation/test split and a fixed random seed of 2025 were adopted, and the base-learner predictions were averaged to obtain the final output.

### 3.4. Gradient Boosting Regression Tree Algorithm

Gradient boosting regression tree (GBRT) is an ensemble learning algorithm based on the Boosting principle [31]. GBRT improves model performance in a stage-wise manner by successively building regression trees to compensate for the unexplained errors generated in earlier learning steps. Owing to its high accuracy and strong generalization ability, GBRT can effectively handle nonlinear feature relationships and complex variable interactions, and has demonstrated excellent fitting performance and robustness in material-property prediction [32].

The GBRT model employed 500 regression trees trained using LSBoost with a learning rate of 0.05. The maximum number of splits and minimum leaf size were set to 25 and 5, respectively. A 70:15:15 train/validation/test split and a fixed random seed of 2025 were adopted.

### 3.5. Development of Prediction Models for SSC

Using the above four models for predicting the compressive strength of SSC, a unified data preprocessing procedure and a consistent external data-splitting strategy were adopted for the entire dataset. Specifically, all predictive models used five input variables as input features, namely curing age (Age), water-to-cement ratio (W/C), sea-sand-to-total-aggregate ratio (S/G), total-aggregate-to-cement ratio (A/B), and superplasticizer-to-cement ratio (SP/C), while the compressive strength of SSC was taken as the output variable. The latter three ratio-based variables were derived from the corresponding raw mixture parameters in the database. The same five input variables were also used for the subsequent SHAP analysis, and no separate model retraining was performed specifically for SHAP interpretation. Before model training, the input variables were Winsorized at the 1st and 99th percentiles and subsequently standardized using Z-score normalization. The Winsorization thresholds and normalization parameters were determined exclusively from the training set and then applied unchanged to the validation and testing sets. A fixed external data split with a ratio of 70:15:15 was adopted for all models to ensure a consistent basis for performance comparison. Based on this data framework, four representative machine-learning models were established. All machine-learning models and SHAP analyses were implemented using MATLAB R2022b.

The detailed parameters of these samples are listed in Table 3.

### 3.6. Performance Evaluation Metrics for Machine-Learning Models

The prediction accuracy of each model was assessed using multiple statistical indicators: the Pearson correlation coefficient (R), coefficient of determination (*R*^2^), mean square error (MSE), root mean square error (*R*_MSE_), and mean absolute error (MAE). Their formulations are given in Table 4 [37,38]. To provide a more comprehensive evaluation of prediction bias and error distribution, the mean prediction ratio, error dispersion, and maximum error were also considered. The mean prediction ratio reflects the overall agreement between predicted and observed values, with a value closer to 1 indicating better prediction consistency. Lower error dispersion and maximum error values indicate smaller prediction variability and extreme deviations, respectively. In general, a high-accuracy predictive model is characterized by lower MSE, RMSE, and MAE values and higher R and R^2^ values.

*n* represents the total number of samples; *x_i_* and *y_i_* denote the observed and predicted values, respectively; *x*_m_ and *y*_m_ are the corresponding mean values of the observed and predicted data.

## 4. Results and Discussion

### 4.1. Basic Mechanical Properties of SSC

#### 4.1.1. Cube Compressive Strength of SSC

**Figure 4:**
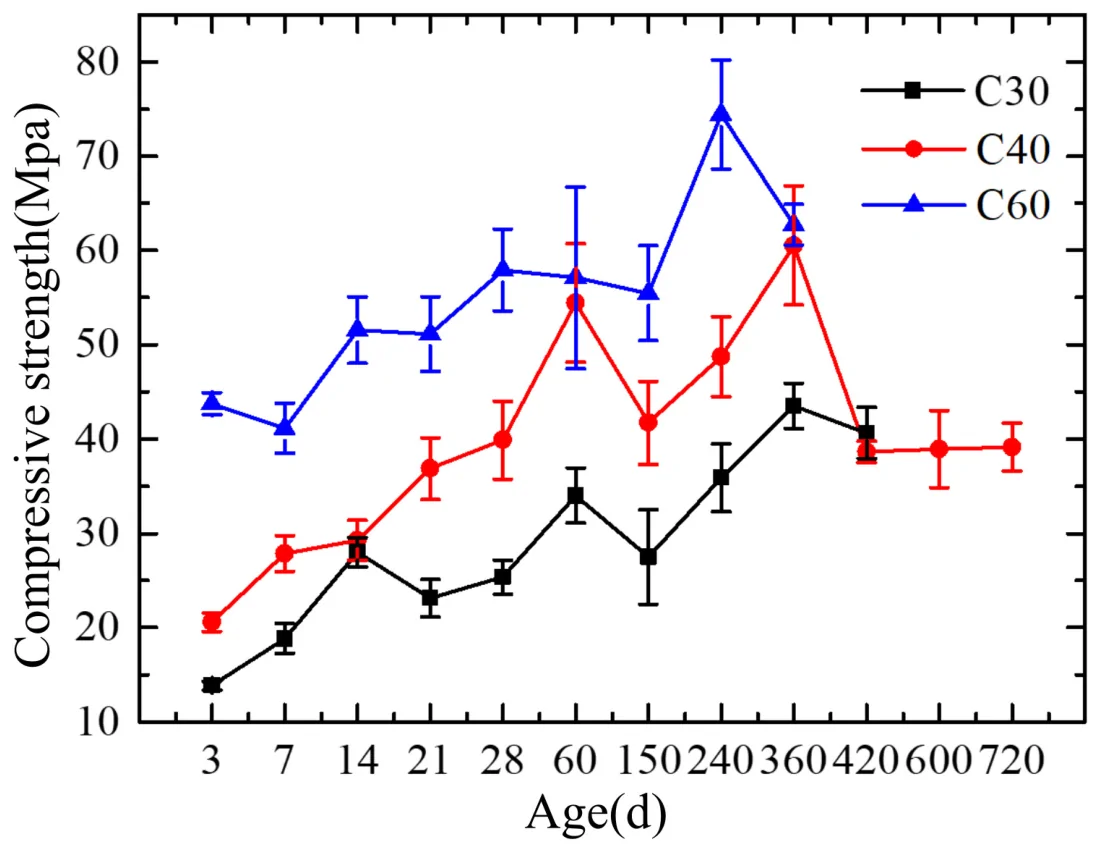
Development of Cube Compressive Strength of SSC.

Figure 4 presents the age-dependent variation in the compressive strength of SSC. The strength dispersion of all strength grades increased after 28 d. For the C30 and C40 mixtures, moderate strength reductions were observed after 60 d and again after 360 d, whereas the C60 mixture showed decreases after 28 d and 240 d. This strength decline may be associated with the dissolution and leaching of hydration products and salt crystallization in the pore network [39], while the possible influence of variations in the unrecorded laboratory temperature and humidity after 28 d cannot be excluded. Similar phenomena have also been reported by Younis et al. [40]. For C40 SSC, the strength at 720 d was substantially lower than the 360 d peak and lower than the value at 150 d, while remaining close to that at 420 d. This behavior further indicates a non-monotonic long-term strength evolution in SSC.

The observed fluctuations may result from the competition between continued hydration and deterioration-related processes. Continued hydration can generate additional hydration products and refine the pore structure, contributing to partial strength recovery [7], whereas the dissolution and leaching of hydration products and salt crystallization may locally weaken the matrix [39,40]. At very long curing ages, such as 720 d, the partial stabilization of C40 SSC may indicate that these opposing processes gradually approach a dynamic balance rather than continuous deterioration.

The effects of seawater and sea sand on the long-term performance of concrete are not limited to strength development. Dissolved ions in seawater can modify cement hydration and may contribute to accelerated strength development at early ages [7,20,33]. However, the later-age evolution is more complex. Previous studies have reported reductions in SSC compressive strength after prolonged curing, accompanied by changes in hydration products and microstructure [20,33]. The leaching of hydration products and the accumulation or crystallization of salts within the pore structure may further contribute to the strength fluctuations observed at later ages [39,40]. In addition, the high chloride content introduced by seawater and sea sand represents an important durability concern for conventionally reinforced concrete, because chloride ingress can increase the risk of steel reinforcement corrosion [2,7,41]. Therefore, the mechanical behavior observed in the present unreinforced SSC specimens should not be interpreted as direct evidence of the long-term durability of reinforced SSC structures. Further evaluation of chloride transport, reinforcement corrosion, and other durability-related properties is required before broader application of SSC in marine structures.

**Figure 5:**
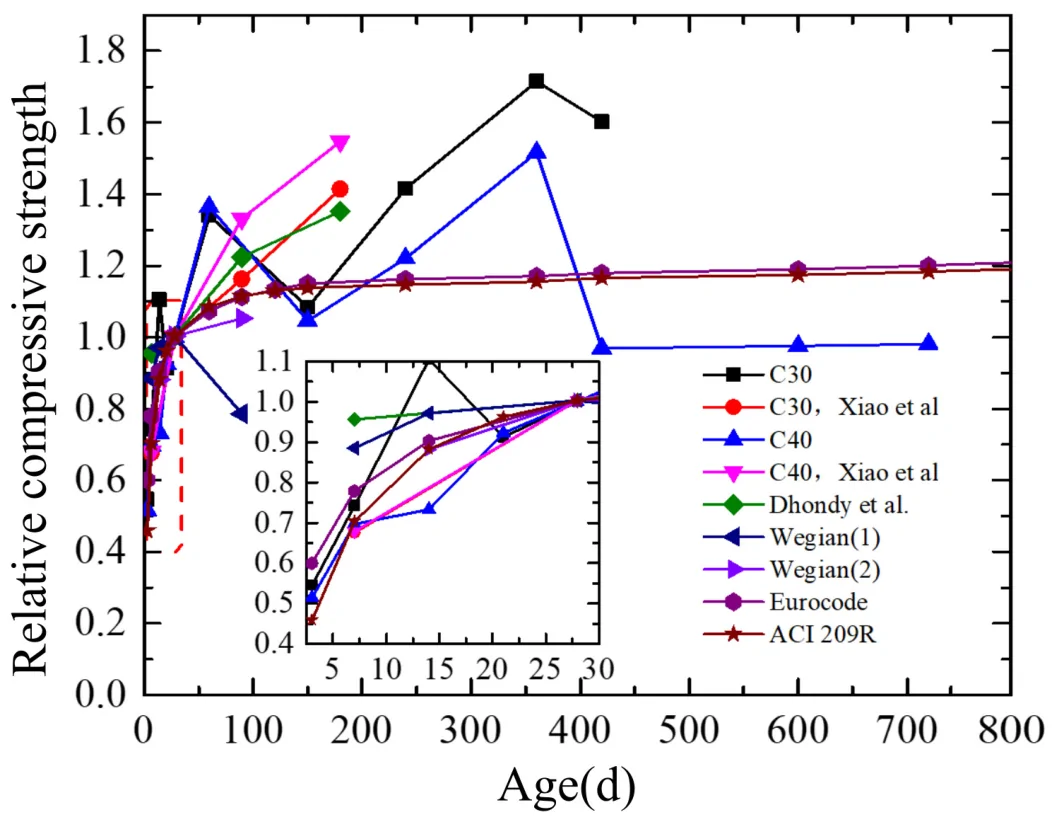
Comparison of compressive strength development of SSC30 and SSC40 with experimental data from Xiao et al. [33], Dhondy et al. [44], and Wegian [39], and predictions from EN 1992-1-1 [42] and ACI 209R [43]. The red dashed box indicates the region enlarged in the inset.

Figure 5 further compares the compressive strength of C30 and C40 SSC with literature data and code-based predictions. The EN 1992-1-1 model [42] overestimated strength development before 7 d, whereas ACI 209R [43] underestimated the early-age strength. Some published studies, such as those of Xiao et al. and Dhondy et al. [33,44], showed a continued increase in strength after 28 d, while Wegian [39] observed a slowdown or even a decline after 90 d. By contrast, the current results exhibit a markedly different trend. After an initial rise of over 30% at 60 days, the strength of C30 and C40 dropped by roughly 30% at 150 days, only to rebound with an increase of over 45% by 360 days. Subsequently, between 360 and 420 days, the compressive strength declined again, decreasing by about 6.6% for C30 and 36.1% for C40. This pattern suggests that the long-term strength evolution of seawater sea-sand systems is substantially more complex than that of conventional concrete. Moreover, the 720 d data for C40 indicate that strength deterioration is not necessarily continuous.

**Figure 6:**
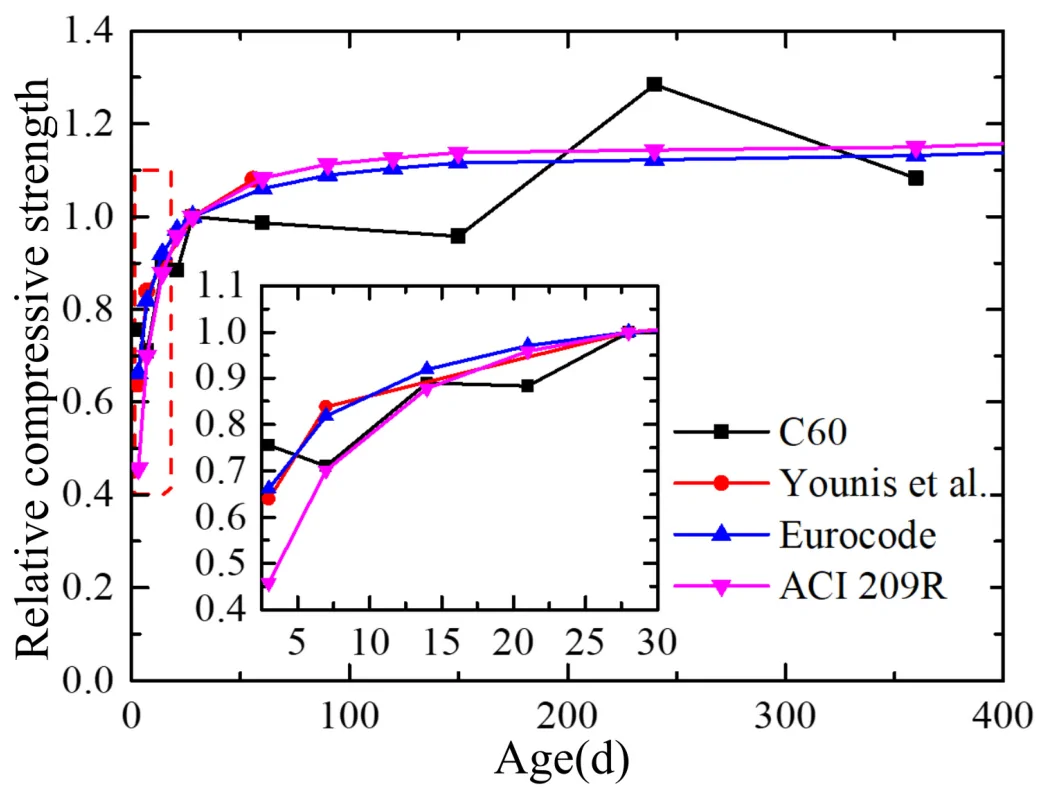
Comparison of SSC60 compressive strength development with experimental data from Younis et al. [40] and predictions from EN 1992-1-1 [42] and ACI 209R [43]. The red dashed box indicates the region enlarged in the inset.

Figure 6 compares the compressive strength development of C60 SSC with previously reported data [40] and code-based predictions. Both design-code curves underestimated the strength development within the first 3 d. At 28 d and 240 d, the C60 SSC in this study showed a decline of no more than 20%, which was inconsistent with the code-based fitted curves; however, the published results agreed well with EN 1992-1-1. Overall, these comparisons reveal marked differences in the long-term strength evolution of SSC from different sources, indicating that SSC strength development is jointly controlled by material composition, ionic environment, and hydration pathway, and is therefore more complex than that of ordinary concrete.

#### 4.1.2. Axial Compressive Strength of SSC

The average ratios of axial compressive strength to cube compressive strength for SSC were compared with the values specified in GB 50010-2010 [45] and ACI 318 [46], as listed in Table 5.

**Figure 7:**
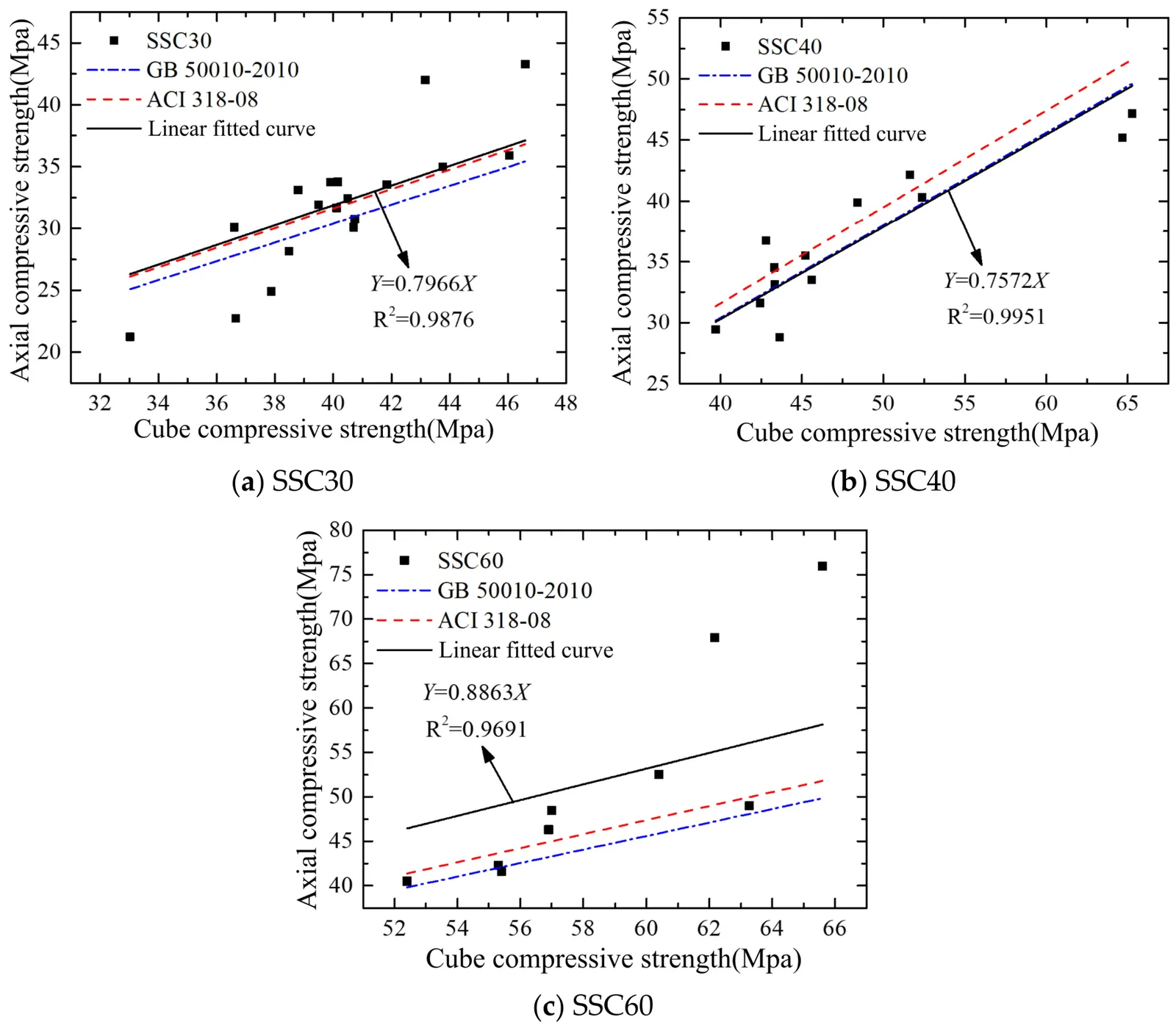
Linear fitting curves of axial and cube compressive strengths of SSC: (**a**) SSC30; (**b**) SSC40; (**c**) SSC60.

The ratios range from 0.74 to 1.04, with deviations of −2% to 30% relative to the value specified in GB 50010. Figure 7 presents the linear fitting relationships for SSC with different strength grades, and the corresponding fitting equations are given in Equation (1). For SSC30 in Figure 7a, after incorporating 15 literature data sets reported by Xiao et al., Geng et al., and Lin et al. [33,34,35], the fitted coefficient was obtained as k_1_ = 0.7966 with R^2^ = 0.9876, and the resulting ratio was in good agreement with the value specified in ACI 318. For SSC40 in Figure 7b, fitting of 13 data sets yielded k_2_ = 0.7572 with R^2^ = 0.9951, which was consistent with the ratio specified in GB 50010. For SSC60 in Figure 7c, the fitted coefficient was k_3_ = 0.8863 with R^2^ = 0.9691. The greater discrepancy between the fitted coefficient and the code-based prediction may result from the relatively narrow data basis used for SSC60 fitting. Overall, the fitted curves for all three strength grades exhibited high coefficients of determination, indicating that the proposed linear models can effectively capture the correlation between axial and cube compressive strengths in SSC.

*f_c_* = *kf_cu_* (1)

#### 4.1.3. Elastic Modulus of SSC

The empirical forms recommended by GB 50010 and ACI 318-02 were used to establish the relationship between elastic modulus and axial compressive strength, and the corresponding fitting equations are presented in Equations (2) and (3). Since the axial compressive strength and elastic modulus were obtained under the same testing conditions in this study, Equation (1) was applied to transform the cube compressive strength *f*_cu_ into the axial compressive strength, *f*_c_. In this way, a quantitative correlation between elastic modulus and axial compressive strength was established.

(2) $$\mathrm{GB}\;50010\;\mathrm{equation}\;\mathrm{form}:E_{c}=\frac{10^{5}}{a+\frac{b}{f_{c}}}$$

(3) $$\mathrm{ACI}\;318\text{-}02\;\mathrm{equation}\;\mathrm{form}:E_{c}=c\;f_{c}^{d}$$

**Figure 8:**
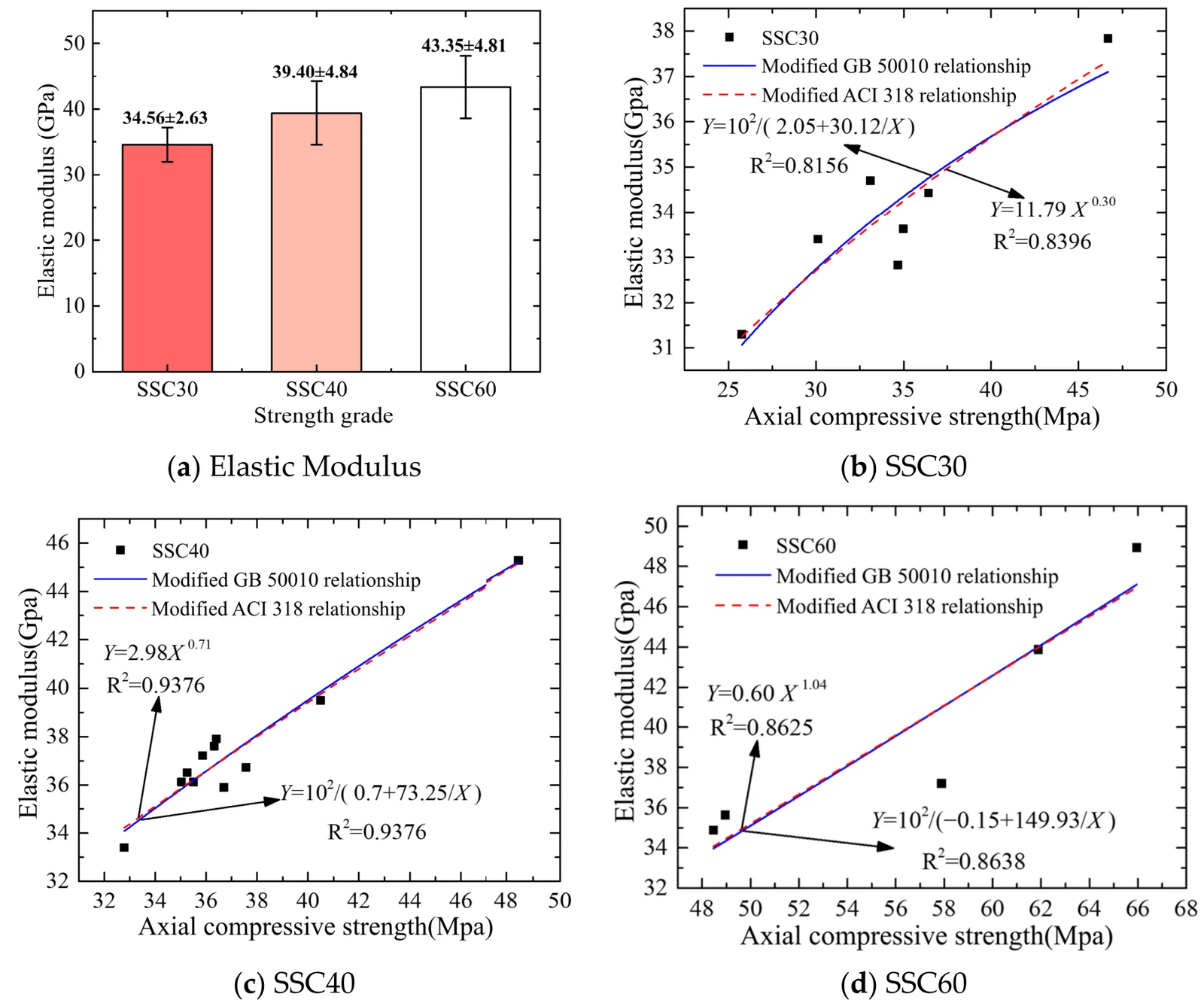
Elastic modulus and its relationship with axial compressive strength of SSC: (**a**) elastic modulus; (**b**) SSC30; (**c**) SSC40; (**d**) SSC60.

The measured elastic modulus values and the corresponding fitting relationships are presented in Figure 8. For SSC30, Figure 8b combines three data sets from the present study with four data sets reported by Xiao et al., Lin, and Geng et al. [33,34,35]. The fitted parameters were obtained as a_1_ = 2.05, b_1_ = 30.12 with R^2^ = 0.8156, and c_1_ = 11.79, d_1_ = 0.30 with R^2^ = 0.8396. For SSC40, Figure 8c incorporates eight data sets from Xiao et al., Zhu et al., and Geng et al. [33,35,36], yielding a_2_ = 0.7, b_2_ = 73.25 with R^2^ = 0.9376, c_2_ = 2.98, d_2_ = 0.71 with R^2^ = 0.9376. For SSC60, Figure 8d uses three data sets from the present study together with two data sets reported by Lin [34], resulting in a_3_ = −0.15, b_3_ = 149.93 with R^2^ = 0.8638, and c_3_ = 0.60, d_3_ = 1.04 with R^2^ = 0.8625. Overall, all coefficients of determination exceeded 0.8, indicating that the proposed relationships can reasonably capture the correlation between elastic modulus and axial compressive strength in SSC.

### 4.2. Microstructural Analysis of SSC

**Figure 9:**
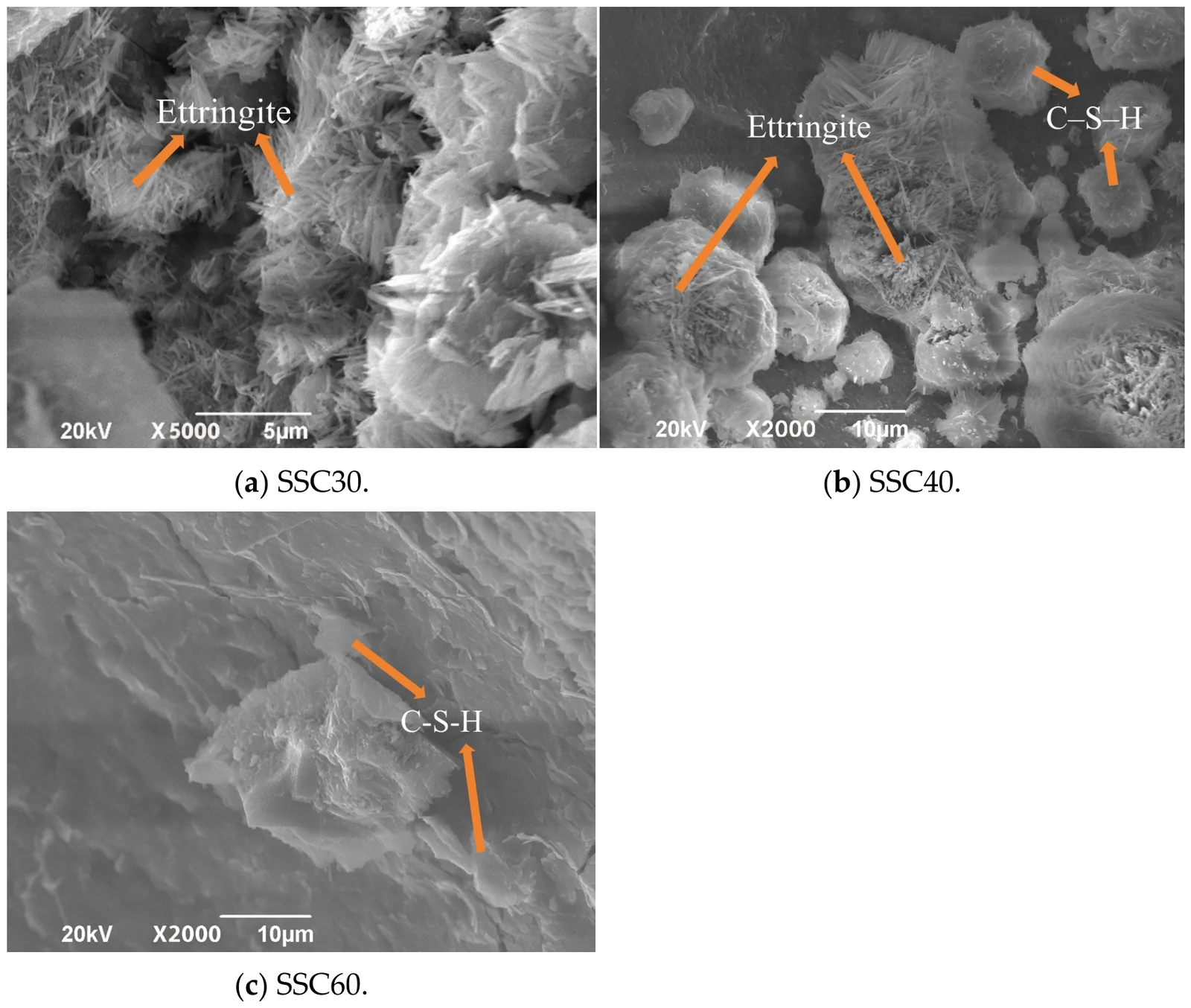
SEM images of SSC hydration-product morphologies at 28 d: (**a**) SSC30, ×5000; (**b**) SSC40, ×2000; (**c**) SSC60, ×2000.

The microstructure of SSC30 is shown in Figure 9a. Needle-like products morphologically consistent with ettringite were *observed* in SSC30, together with a relatively heterogeneous matrix morphology. These long, slender, needle-like products grew in multiple directions along the gel-like matrix surface. Previous studies have reported that seawater can promote calcium-aluminate hydration and favor ettringite formation [47]. The additional ettringite can induce expansion in concrete and thus reduce its autogenous shrinkage [47,48]. Subsequently, the continuous formation of C–S–H gel further refined the pore structure.

The microstructure of cement hydration products in SSC40 is presented in Figure 9b. Compared with the field of view of SSC30, fewer needle-like products were observed in the SSC40 image. This morphological difference may be associated with the lower W/C ratio and other simultaneous changes in mixture composition, although the present SEM observations alone cannot isolate their individual effects. As shown in Figure 9c, the observed field of SSC60 appeared relatively compact; however, this local morphology alone cannot be used to explain its higher strength relative to SSC30 and SSC40. Since the SEM observations were conducted only at 28 d, the present microstructural analysis is primarily qualitative and cannot establish a quantitative relationship between microstructural evolution and compressive-strength development over the full curing period. Quantitative microstructural characterization at multiple curing ages is therefore required to establish such relationships in future studies. The phase annotations in Figure 9 are based on characteristic SEM morphologies and literature reports and therefore should be regarded as morphology-based identification rather than independent phase confirmation.

### 4.3. Predictive Models for the Compressive Strength of SSC

**Figure 10:**
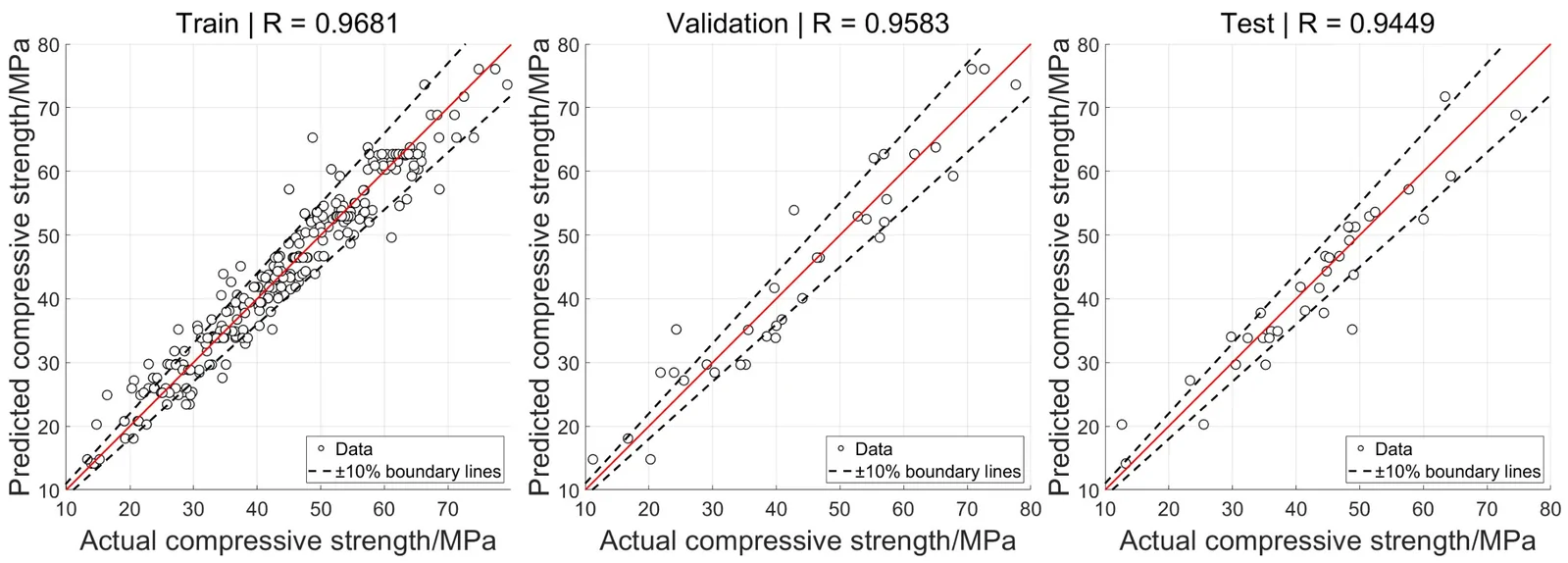
Comparison between BP predictions and experimental values.

**Figure 11:**
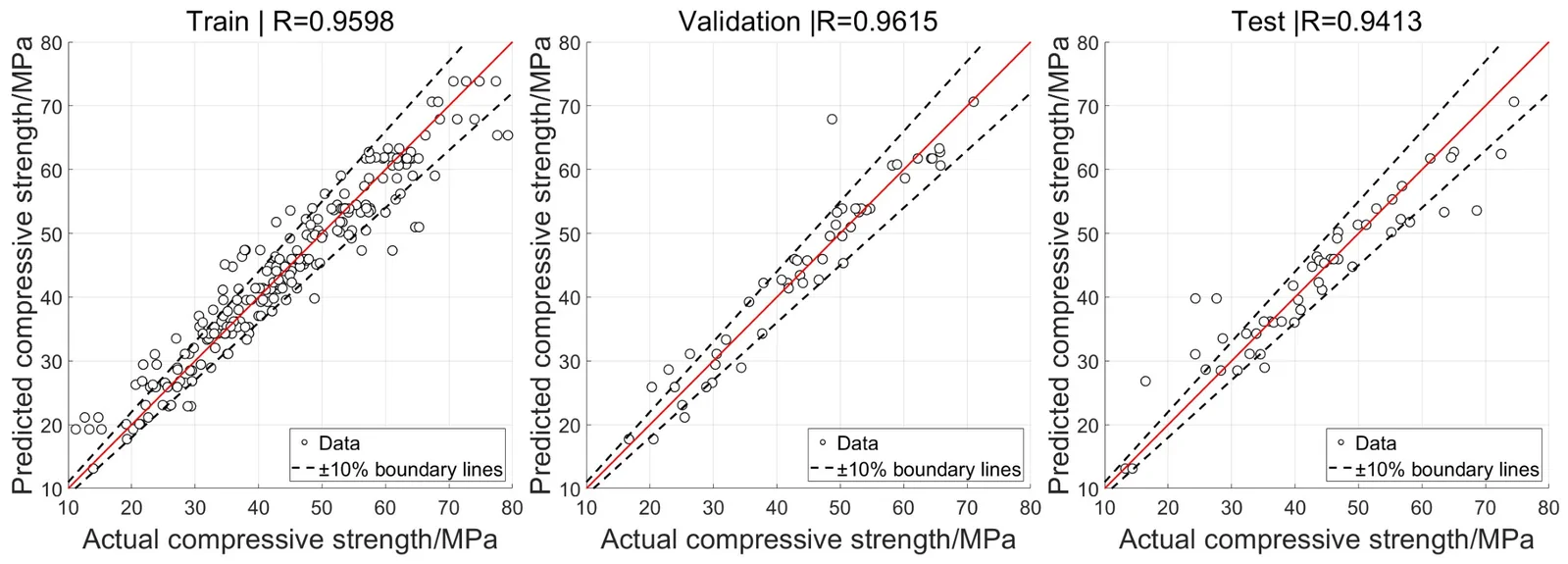
Comparison between GA-BP predictions and experimental values.

**Figure 12:**
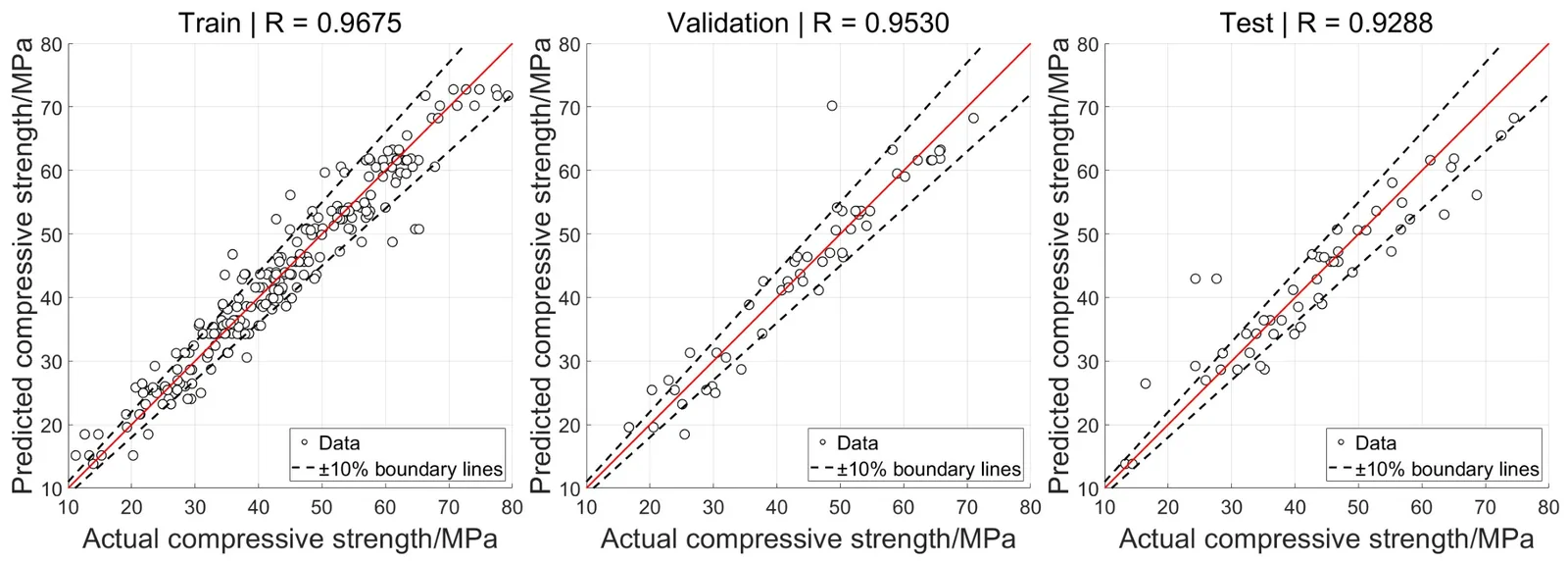
Comparison between Bagging-BP predictions and experimental values.

**Figure 13:**
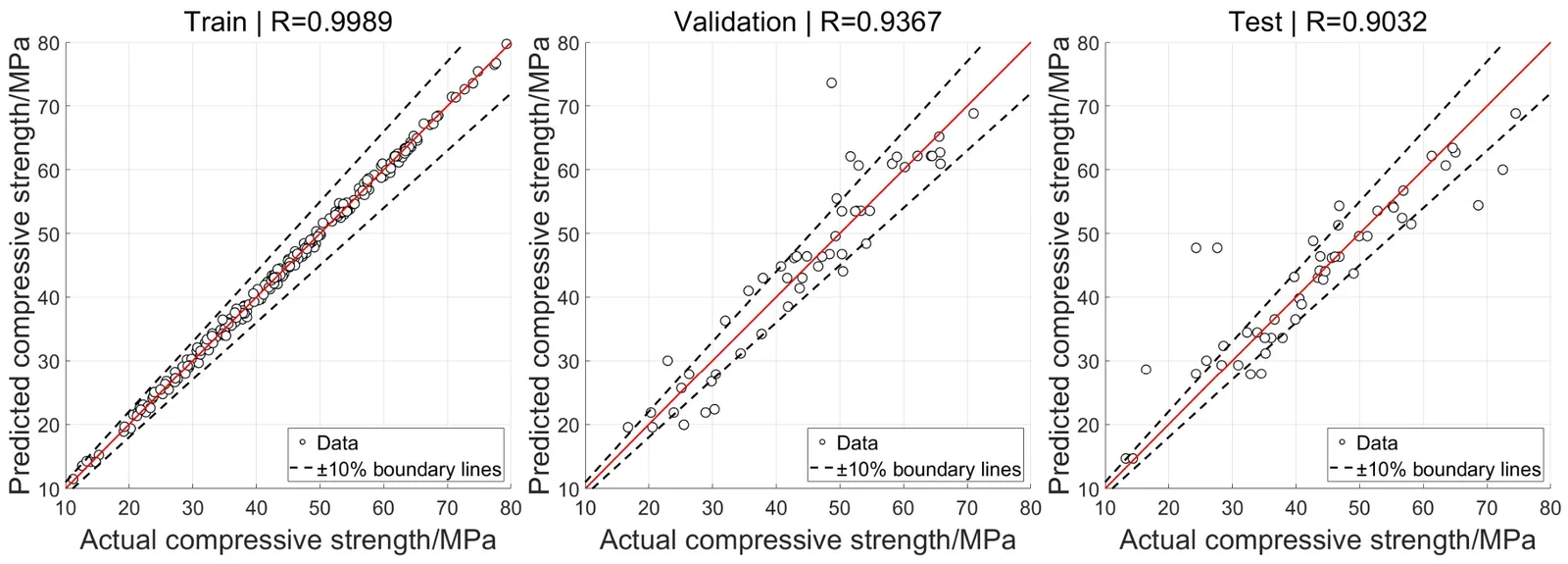
Comparison between GBRT predictions and experimental values.

The predictive performance of the four models, namely BP, GA-BP, Bagging-BP, and gradient boosting regression tree (GBRT), is summarized in Table 6 and Figure 10, Figure 11, Figure 12 and Figure 13. Based on the feature system established in this study, the predictive performance of the four machine-learning methods was compared on the training, validation, and test sets using multiple statistical indicators.

From the training results, the BP neural network was capable of capturing the nonlinear development trend of SSC compressive strength, which is consistent with earlier studies on artificial neural network modeling of concrete strength [49,50]. For the training set, the RMSE values of the BP, GA-BP, Bagging-BP, and GBRT models were 4.0012, 4.0255, 3.5948, and 0.6793, respectively. Although the GA-BP model exhibited a slightly higher RMSE during training, its genetic-algorithm-based enhancement of the search for initial weights and thresholds laid the foundation for improved generalization in subsequent prediction stages [49]. The mean prediction ratios of all four models were close to 1.0, while GBRT exhibited the lowest error dispersion (1.9%), further confirming its strong fitting capability on the training set.

On the validation set, the RMSE values of the four models were 4.9672 (BP), 3.8321 (GA-BP), 4.2534 (Bagging-BP), and 5.1587 (GBRT). Among them, GA-BP produced the smallest RMSE, indicating favorable validation performance under the adopted data split. Previous studies have likewise shown that GA-based global optimization can enhance the stability of BP networks and reduce their tendency toward overfitting [49,51]. The Bagging-BP model also exhibited robust performance during validation, and its relatively low fluctuation was consistent with existing findings that Bagging ensembles can improve model robustness in concrete-property prediction [30]. The mean prediction ratios of all models remained close to 1.0 on the validation set, while GA-BP showed the lowest error dispersion (10.2%).

On the test set, the RMSE values of the BP, Bagging-BP, and GBRT models were 6.2296, 5.4546, and 6.1420 MPa, respectively, whereas the GA-BP model achieved an RMSE of 5.0234 MPa, showing favorable generalization performance. The corresponding MAE values of BP, Bagging-BP, GA-BP, and GBRT were 4.1775, 3.7848, 3.3680, and 3.8069 MPa, respectively, while their R^2^ values were 0.8080, 0.8528, 0.8751, and 0.8133. These results indicate that GA-BP achieved the most favorable overall predictive performance on the test set under the adopted data split. The mean prediction ratios of all four models remained close to 1.0 on the test set, while BP showed the lowest error dispersion (12.4%). In contrast, although GBRT performed exceptionally well on the training set, its performance declined considerably on the test set, with R decreasing from 0.9989 to 0.9032 and RMSE increasing from 0.6793 to 6.1420 MPa. Its test-set error dispersion reached 21.5%, indicating a tendency toward overfitting and reduced prediction stability for unseen samples. This trend is consistent with the tendency of gradient boosting algorithms to exhibit overfitting when the sample size is limited [52].

Overall, all four models demonstrated good predictive capability in capturing the feature–strength relationship, although their strengths differed. The BP model was effective in capturing nonlinear relationships while maintaining stable performance. GA-BP enhanced generalization through global optimization [49,51]. Bagging-BP, by relying on an ensemble strategy, showed relatively stable predictive performance under the adopted data split. GBRT, in contrast, exhibited the most prominent fitting capability and can serve as a valuable reference for structured feature mining and model integration [52]. These results further indicate that, for multi-source and multivariable systems such as SSC, achieving high-accuracy prediction requires simultaneous consideration of both fitting capability and generalization performance.

The current models were developed using an individual-record-level random split, and leave-one-source-out validation has not yet been conducted, which may introduce potential information leakage. Nevertheless, the compiled database covers SSC compressive strengths of approximately 11.2–79.3 MPa from several experimental sources. Within this domain, the GA-BP model may be used for preliminary strength prediction and mix screening; however, its applicability should be limited to the data range represented by the current database, and further group-wise and source-level validation is required before application to substantially different SSC systems.

### 4.4. Interpretability Analysis Based on SHAP Method

The SHAP method [53,54] is a model-interpretation approach designed to quantitatively characterize the prediction mechanism of machine-learning models. The calculated SHAP values allow the effect of each input variable on the model output to be quantitatively assessed at both the sample and overall dataset levels. Therefore, SHAP analysis helps clarify the decision-making mechanism of the model and improves the transparency of the prediction results.

**Figure 14:**
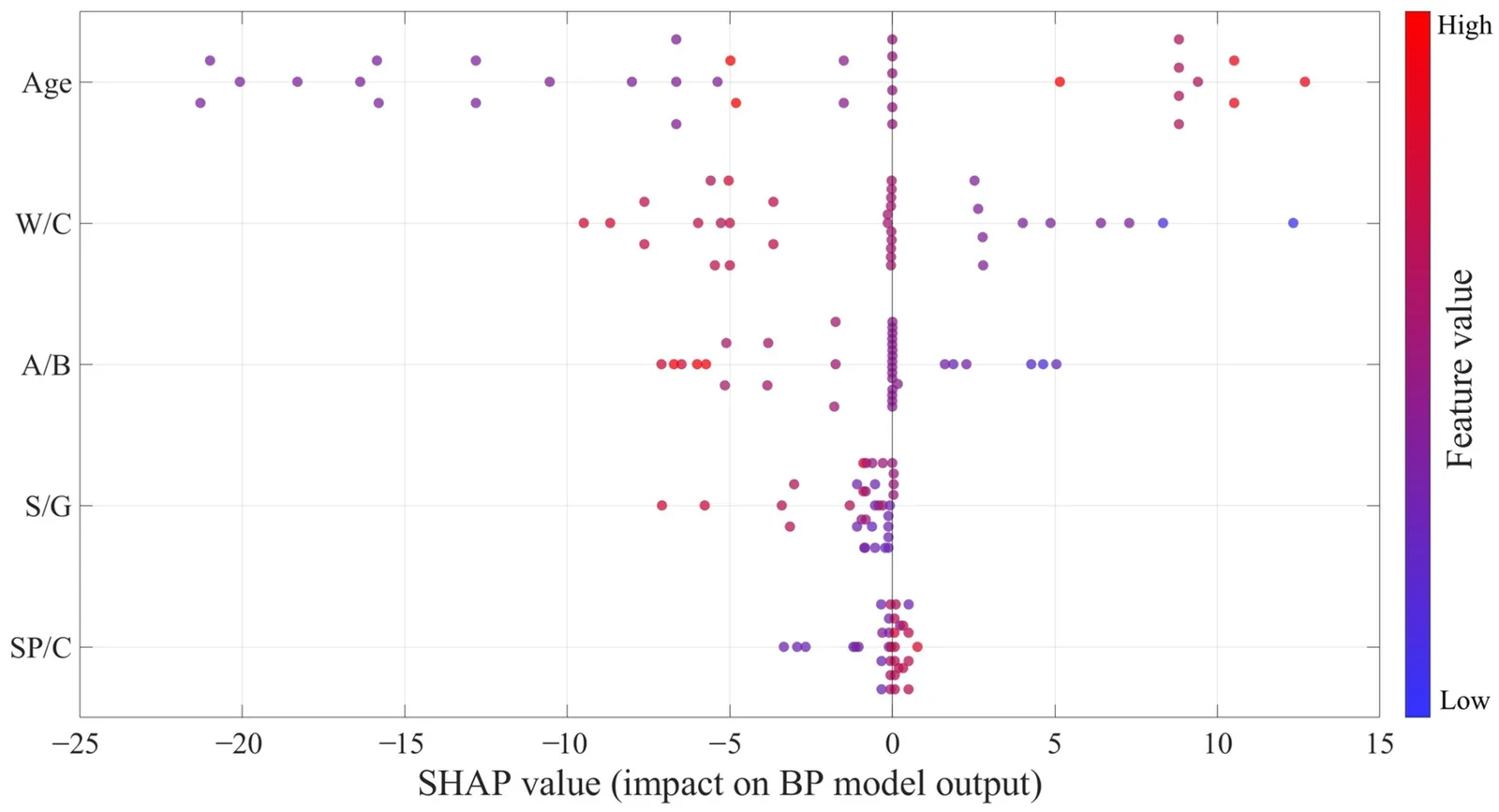
SHAP analysis results of the BP model.

**Figure 15:**
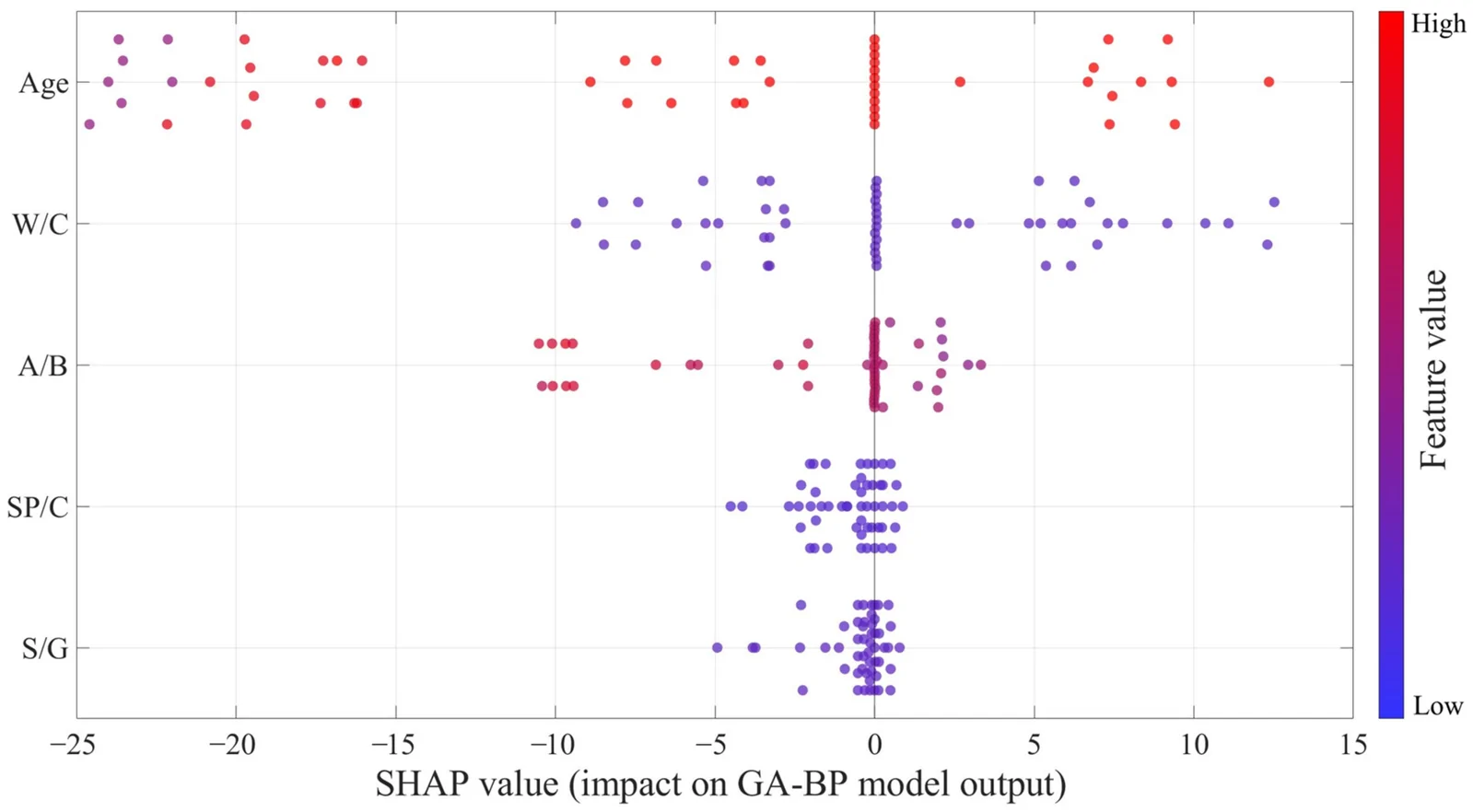
SHAP analysis results of the GA-BP model.

**Figure 16:**
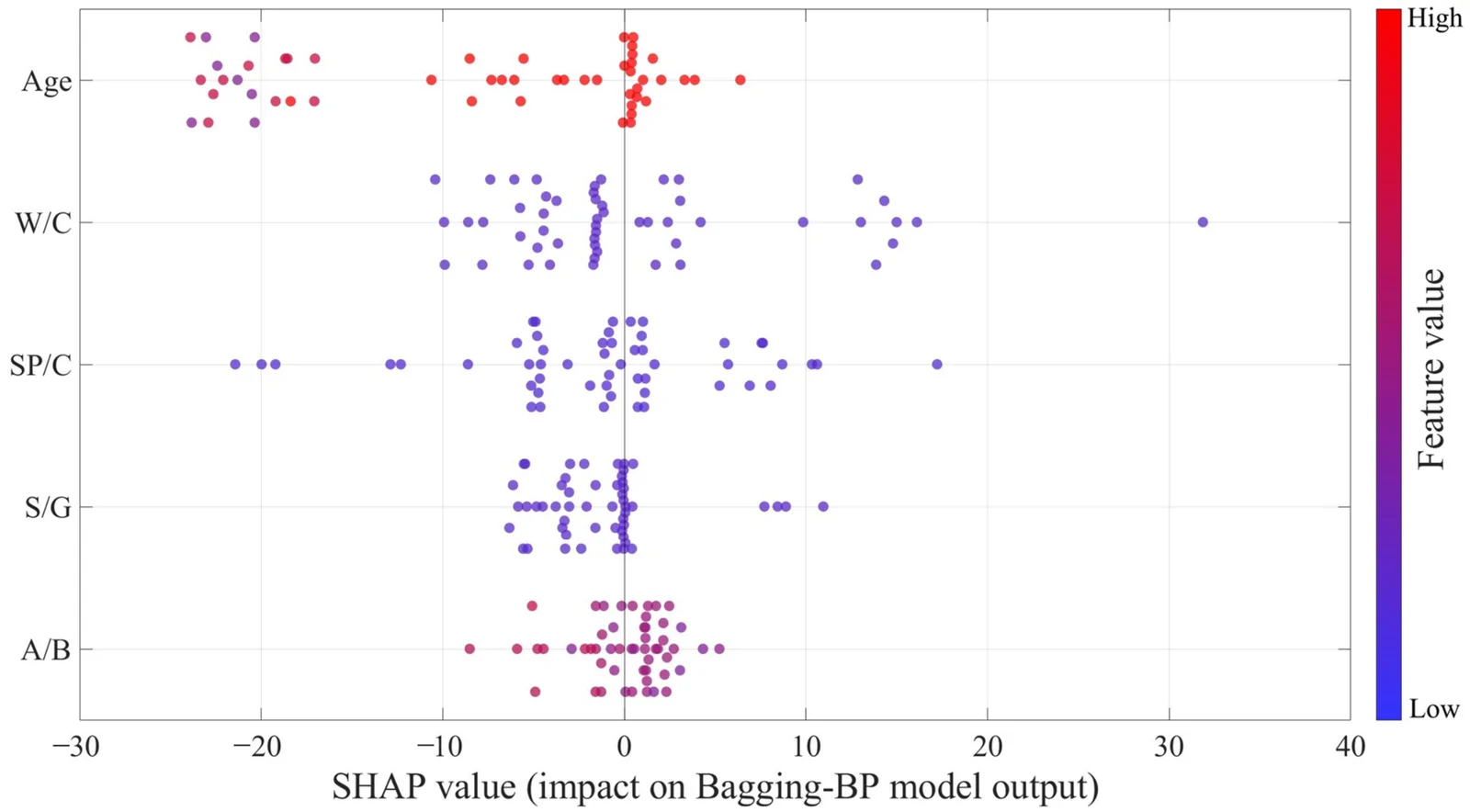
SHAP analysis results of the Bagging-BP model.

**Figure 17:**
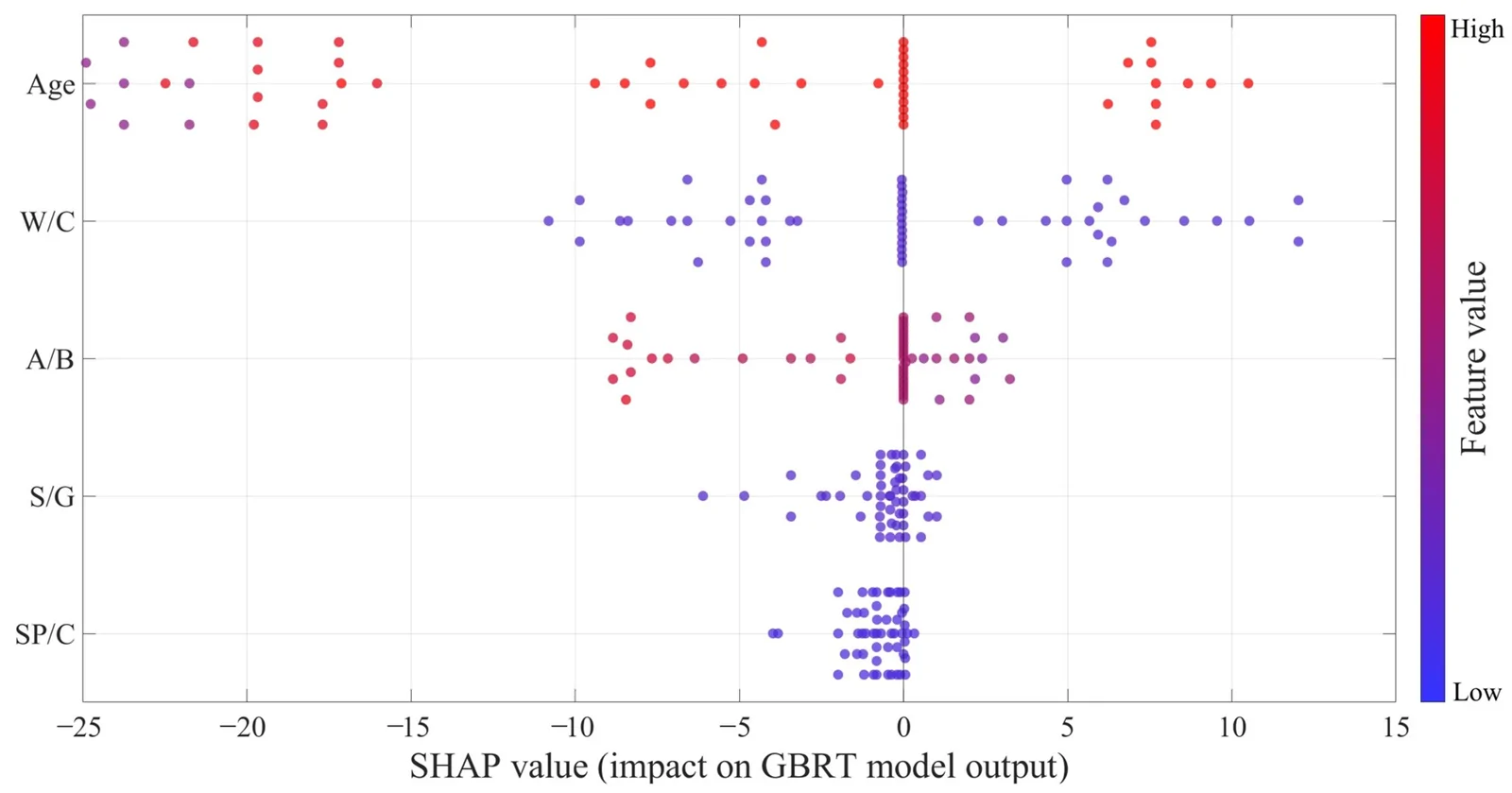
SHAP analysis results of the GBRT model.

**Figure 18:**
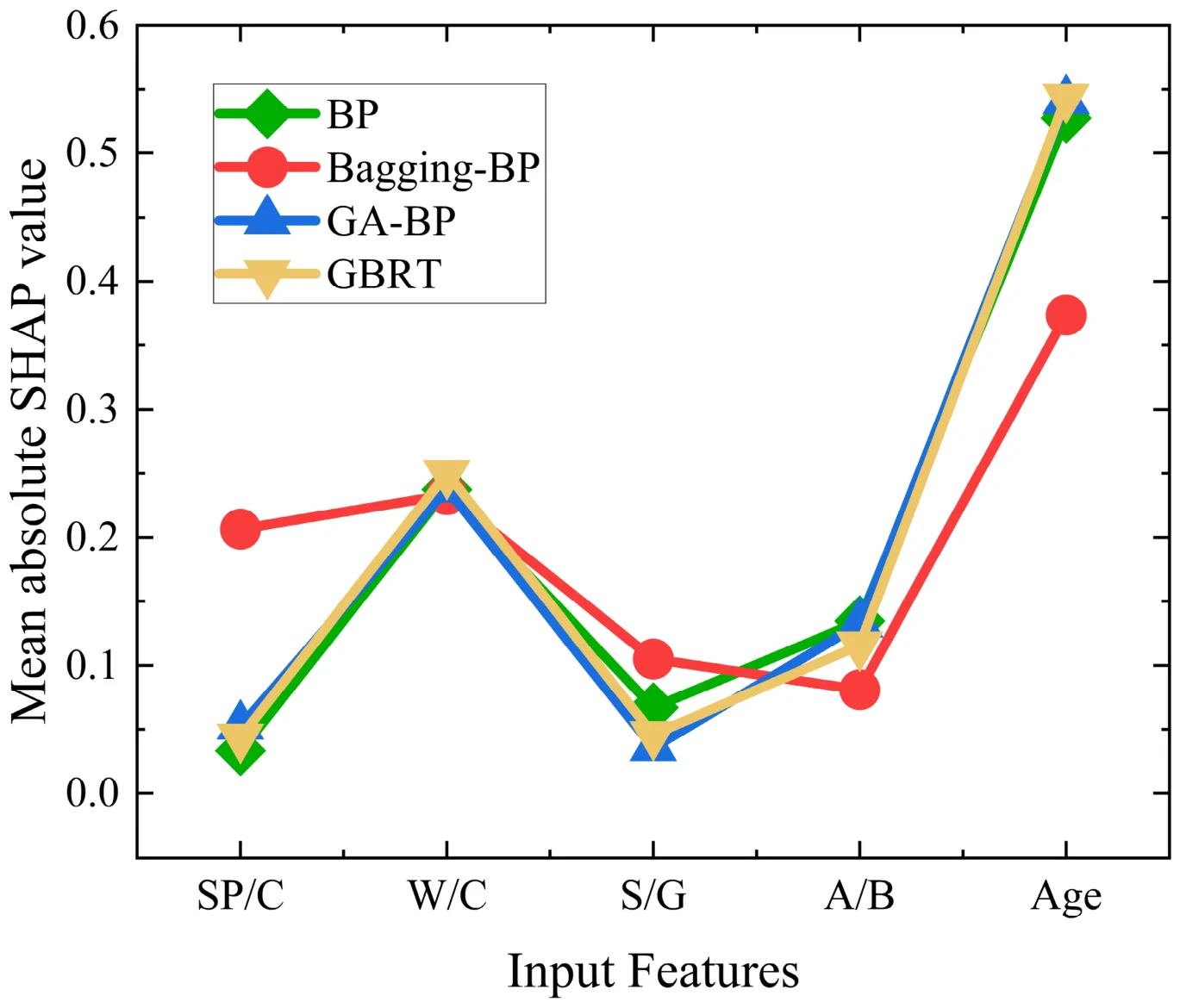
Comparison of SHAP analysis results among different models.

In this study, Age, W/C, A/B, S/G, and SP/C were selected as input features for SHAP analysis. For all four models, the training set was used as the background reference, while the validation set was used as the set of samples to be explained. SHAP values were estimated using a Monte Carlo permutation-based procedure. For Bagging-BP, SHAP analysis was applied directly to the final ensemble prediction function. For each perturbed sample, the predictions of all 15 BP base learners were averaged to obtain the ensemble output before calculating the marginal contribution of each feature. Figure 14, Figure 15, Figure 16, Figure 17 and Figure 18 present the SHAP interpretation results for SSC strength prediction using the BP, GA-BP, Bagging-BP, and GBRT models. Specifically, Figure 14, Figure 15, Figure 16 and Figure 17 show the SHAP scatter plots for each model. Each point in these plots corresponds to the SHAP value of an individual sample for a given feature. In this SHAP-based visualization, the chromatic variation in each dot reflects the relative magnitude of its associated input variable; blue tones indicate lower values, whereas red tones indicate higher values. The *x*-axis location of each sample point represents its contribution to the model output. SHAP values on opposite sides of zero indicate contrasting effects, while points farther from zero suggest a larger contribution magnitude. Figure 18 compares the overall importance of the input variables across the different models based on the mean absolute SHAP values.

As illustrated in Figure 18, the models exhibited strong agreement in identifying the key influencing factors. Age emerged as the most influential factor across all models. Its SHAP values were predominantly positive and increased with longer curing time, indicating a favorable effect on strength development. This behavior is mainly related to the continuous hydration of cement. During this process, hydration products gradually fill the internal pores and densify the microstructure, thereby improving the compressive strength of SSC.

The W/C ratio played an unfavorable role in SSC strength prediction. With an increase in W/C, the SHAP values shifted overall toward the negative direction, indicating that an excessively high W/C reduces matrix compactness and is unfavorable for strength development. Conversely, reducing W/C helps decrease pore content and enhance the compactness of the cementitious matrix.

The dominant effects of Age and W/C are consistent with conventional concrete, where continued hydration and lower porosity largely govern strength development. SSC-specific factors, including chloride content, sea-sand salinity, and mineral composition, may also influence hydration and microstructure. However, these variables were not consistently available in the compiled database and were not included as model inputs; therefore, their individual effects and interactions with Age and W/C cannot be quantified by the present SHAP analysis.

The influence of A/B and S/G, though secondary, remained non-negligible. The SHAP distributions indicate that a larger A/B generally corresponded to negative SHAP values, suggesting that insufficient paste weakens the bonding effect between aggregates and thus reduces strength. In contrast, a moderate increase in S/G helps optimize aggregate gradation and fill the voids between aggregates, causing the SHAP values to shift overall in the positive direction and thereby contributing to compressive-strength development to a certain degree.

By comparison, SP/C generally showed a relatively limited contribution in the BP, GA-BP, and GBRT models, whereas the Bagging-BP model assigned it a comparatively higher importance.

Overall, the models showed good agreement in identifying Age and W/C as the two most influential features, while the relative importance of A/B, S/G, and SP/C showed some model-dependent variation. In particular, the Bagging-BP model followed the order Age > W/C > SP/C > S/G > A/B. This indicates that although the models consistently identified the dominant effects of Age and W/C, some differences existed in their evaluation of the secondary mixture-related variables.

## 5. Conclusions

This study systematically elucidated the mechanical evolution, microstructural characteristics, and data-driven strength prediction of seawater sea-sand concrete (SSC). The principal conclusions are as follows:

(1) SSC exhibits pronounced age-dependent mechanical evolution. The experimental results generally showed rapid strength development at early ages, followed by non-monotonic fluctuations at middle and later ages. The evolution pattern varied with strength grade. C30 and C40 SSC exhibited stagewise peaks near 60 d and 360 d, followed by reductions at 150 d and 420 d. C60 SSC showed more marked fluctuations, with descending intervals already emerging after 28 d and 240 d. For C40, the strength at 720 d was substantially lower than the 360 d peak and the 150 d value, but remained close to the 420 d value.
(2) Quantitative strength–elastic modulus relationships were established for C30, C40, and C60 SSC. All fitted models achieved coefficients of determination greater than 0.80, with the C40 model reaching an R^2^ value close to 0.94. This confirms that the proposed relationships can reliably characterize the intrinsic correlation among the mechanical properties of SSC.
(3) Four machine-learning models—BP, Bagging-BP, GA-BP, and GBRT—were established for SSC compressive-strength prediction. Among these models, GA-BP consistently yielded the smallest RMSE values for the validation and test sets, achieving a test-set RMSE of 5.0234 MPa. This demonstrates its favorable predictive performance under the adopted data split.
(4) SHAP-based interpretability analysis identified the key parameters governing the compressive strength of SSC. The models showed generally consistent trends in feature importance. The SHAP distributions showed that curing age exerted the dominant influence, with the water-to-cement ratio ranked second. The effects of the aggregate-to-binder ratio and sand ratio were secondary but still non-negligible. By contrast, SP/C showed a relatively limited contribution in the BP, GA-BP, and GBRT models, whereas the Bagging-BP model assigned it comparatively higher importance.

## Figures and Tables

**Table 1:** Physical properties of sea sand.

Fineness Modulus	Apparent Density (kg/m^3^)	Shell Content (wt%)	Silt Content (%)	Surface-Adhered Chloride Content (wt%)	Surface-Adhered Chloride Ion Content (wt%)
2.31	2549	1.53	1.08	0.13	0.03

**Table 2:** Mix proportions of SSC (kg/m^3^) [20].

Concrete	w/c Ratio	Seawater	Sea Sand	Cement	Crushed Stone	Superplasticizer
C30	0.47	150	829	319 (P.O. 42.5)	1100	0.957
C40	0.40	150	749	375 (P.O. 42.5)	1113	1.125
C60	0.32	174	554	544 (P.O. 52.5)	1100	1.034

**Table 3:** Compressive Strength Data of SSC Samples.

Sample No.	Cement (kg/m^3^)	Water-to-Cement Ratio	Sea Sand (kg/m^3^)	Coarse Aggregate (kg/m^3^)	Superplasticizer (kg/m^3^)	Age (d)	Compressive Strength (MPa)	Source
1~63	319~544	0.32~0.47	544~829	1100~1113	0.957~1.125	3~150	13.3~68.6	Previous study [20]
64~93	319~544	0.32~0.47	544~829	1100~1113	0.957~1.125	240–720	31.2~79.3	Present study
94~157	288~406	0.37~0.52	708~843	1099~1131	2.590~3.650	7~180	11.3~74.0	Xiao et al. [33]
158~212	410~628	0.30~0.50	615~669	1005~1092	1.230~3.420	3~90	20.3~72.5	Lin [34]
213~296	370~463	0.40~0.50	573~682	1113~1164	2.960~6.019	3~360	22.2~74.5	Geng [35]
297~344	411	0.45	596	1158	4.110	7~180	31.2~65.2	Zhu et al. [36]

Note: The detailed information is provided in Table S1 in the Supplementary Materials.

**Table 4:** Statistical metrics.

Statistical Indicators	Formula
Pearson correlation coefficient (R)	$R=\frac{\sum_{i=1}^{n}\;\left(x_{i}-x_{m}\right)\left(y_{i}-y_{m}\right)}{\sqrt{\sum_{i=1}^{n}\;(x_{i}-x_{m}{)}^{2}\sum_{i=1}^{n}\;(y_{i}-y_{m}{)}^{2}}}$
Coefficient of determination (*R*^2^)	$R^{2}=1-\frac{\sum_{i=1}^{n}{\left(y_{i}-x_{i}\right)}^{2}}{\sum_{i=1}^{n}{\left(x_{i}-x_{m}\right)}^{2}}$
Mean squared error (MSE)	$\mathrm{MSE}=\frac{1}{n}\sum_{i=1}^{n}{\left(y_{i}-x_{i}\right)}^{2}$
Root mean square error (*R*_MSE_)	$\mathrm{RMSE}=\sqrt{\frac{1}{n}\sum_{i=1}^{n}\left({\left(y_{i}-x_{i}\right)}^{2}\right)}$
Mean absolute error (MAE)	$\mathrm{MAE}=\frac{1}{n}\sum_{i=1}^{n}\left|y_{i}-x_{i}\right|$

**Table 5:** The ratio of axial and cube compressive strength.

	C30	C40	C60
*f*_c_ (MPa)	38.67 ± 6.90	44.83 ± 2.52	65.45 ± 11.91
*f*_cu_ (MPa)	43.50 ± 2.94	60.54 ± 7.71	62.72 ± 2.65
*f*_c_/*f*_cu_ SSC	0.89	0.74	1.04
*f*_c_/*f*_cu_ GB 50010	0.76	0.76	0.74
*f*_c_/*f*_cu_ ACI 318	0.79	0.79	0.79

**Table 6:** Prediction Results of Compressive Strength of SSC.

	BP Neural Network			Bagging-BP Neural Network			GA-BP Neural Network			GBRT		
	Train	Validation	Test	Train	Validation	Test	Train	Validation	Test	Train	Validation	Test
R	0.9681	0.9583	0.9449	0.9675	0.9530	0.9288	0.9598	0.9615	0.9413	0.9989	0.9367	0.9032
R^2^	0.9204	0.8689	0.8080	0.9358	0.9039	0.8528	0.9195	0.9220	0.8751	0.9977	0.8586	0.8133
MSE (MPa^2^)	16.0099	24.6735	38.8084	12.9227	18.0917	29.7531	16.2046	14.6849	25.2348	0.4615	26.6119	37.7242
RMSE (MPa)	4.0012	4.9672	6.2296	3.5948	4.2534	5.4546	4.0255	3.8321	5.0234	0.6793	5.1587	6.1420
MAE (MPa)	2.8625	3.8868	4.1775	2.6568	2.9012	3.7848	2.9339	2.6226	3.3680	0.5597	3.5140	3.8069
Mean prediction ratio	1.0146	0.9995	1.0093	1.0044	1.0044	1.0102	1.0159	1.0123	1.0177	1.0013	1.0103	1.0355
Error Dispersion (%)	11.6	11.8	12.4	9.5	11.2	18.0	12.1	10.2	16.4	1.9	12.8	21.5
Maximum error (%)	61.4	38.0	36.9	46.3	44.1	76.9	72.3	39.4	63.8	7.3	51.2	96.4

## Data Availability

The original contributions presented in this study are included in the article/Supplementary Material. Further inquiries can be directed to the corresponding author.

## Supplementary Materials

The following supporting information can be downloaded at https://www.mdpi.com/article/10.3390/ma19204423/s1, Table S1: Detailed information on the SSC compressive-strength dataset.
